# Changes in Periodontal Parameters and Microbiome Composition of Periodontal Pocket in Patients with Chronic Inflammatory Diseases Receiving Targeted Anti-Cytokine Therapy

**DOI:** 10.3390/microorganisms12101934

**Published:** 2024-09-24

**Authors:** Juliane Wagner, Luisa Haker, Louisa Mewes, Corinna Bang, Malte Rühlemann, Hendrik Naujokat, Johannes Heinrich Spille, Wolfgang Lieb, Andre Franke, Stefan Schreiber, Matthias Laudes, Christof Dörfer, Jörg Wiltfang, Christian Graetz, Dominik Maria Schulte

**Affiliations:** 1 Department of Oral and Maxillofacial Surgery, University-Hospital Schleswig-Holstein, Campus Kiel, 24105 Kiel, Germany; 2 Cluster of Excellence, Precision Medicine in Inflammation, Christian-Albrechts-University of Kiel, 24105 Kiel, Germany; 3 Department of Prosthodontics, Geriatric Dentistry and Craniomandibular Disorders, Charité-Universitätsmedizin Berlin, Corporate Member of Freie Universität Berlin, Berlin Institute of Health, Humboldt-Universität zu Berlin, 14197 Berlin, Germany; 4 Institute of Clinical Molecular Biology, Christian-Albrechts-University of Kiel, 24105 Kiel, Germany; 5 Institute of Epidemiology, Christian-Albrechts-University of Kiel, 24105 Kiel, Germany; 6 Department of Internal Medicine I, University-Hospital Schleswig-Holstein, Campus Kiel, 24105 Kiel, Germany; 7 Institute of Diabetes and Clinical Metabolic Research, University-Hospital Schleswig-Holstein, Campus Kiel, 24105 Kiel, Germany; 8 Clinic of Conservative Dentistry and Periodontology, University-Hospital Schleswig-Holstein, Campus Kiel, 24105 Kiel, Germany

**Keywords:** chronic inflammation, periodontitis, microbiome, anti-cytokine therapy

## Abstract

Periodontitis is associated with systemic chronic inflammatory diseases. There is limited evidence on the influence of anti-cytokine therapies on the periodontal condition and microbiome in the tooth pocket of such patients, so the aim of this study was to elucidate this issue. In this observational trial, the periodontal status and the gingival crevicular fluid of 13 patients with different chronic inflammatory diseases were obtained before the initiation of anti-cytokine treatment and 14 weeks after. Gingival crevicular fluid was collected for 16S rRNA gene sequencing from a clinically healthy tooth and the deepest measured pocket. The Shannon Diversity Index significantly increased in the deepest pockets of patients (*p* = 0.039). The data showed alterations in the diversity of the subgingival microbiome over the course of the study, implying a shift towards a healthier condition after starting anti-cytokine therapy. Additional investigations are needed to analyze whether the administration of selective biologicals can improve periodontal conditions in patients with or without chronic inflammatory diseases.

## 1. Introduction

Chronic inflammation is known to be provoked by the interaction of human organ interfaces with the environment, leading to dysfunctional conditions. This is assumed to be the cause of many chronic diseases that have become a widespread public health issue, especially in the western hemisphere. Highly prevalent examples of chronic inflammatory diseases (CIDs) include classic inflammatory diseases such as inflammatory bowel disease (IBD), Crohn’s disease, and ulcerative colitis [1,2], as well as rheumatoid arthritis [3], psoriasis, and psoriatic arthritis [4]. In recent years, it has become increasingly clear that other systemic diseases should also be counted as inflammatory diseases. These include atherosclerosis, and thus cardiovascular inflammation [5], and metabolic inflammation [6,7]. Moreover, they are all associated with periodontitis, as well as other conditions [8,9,10,11,12,13,14].

A review by Holmstrup et al. [15] describes shared inflammatory pathways that could lead to a combination of all these diseases, implying that a targeted therapeutic intervention could also improve the clinical outcome of the other related diseases. CIDs represent a central challenge for modern medicine, and treating them is an example of modern precision medicine. Targeted anti-cytokine therapies are applied to modulate or suppress the disturbed immune response. To date, there has been a limited number of studies examining the effects of these specific therapies on the oral interface, particularly oral status and the periodontal microbiome. The aim of this pilot study was to investigate the influence of biological treatment for CID on the periodontal status and the oral microbiome.

Periodontitis is a chronic inflammatory disease of multicausal pathogenesis characterized by dysbiosis of the subgingival biofilm [16,17,18]. As early as the 1960s, it was shown that an accumulation of periodontal biofilm leads to the development of periodontitis [19]. At the end of the 20th century Marsh and Bradshaw developed the biofilm hypothesis, which states that periodontitis develops when a certain number of periodontal pathogens colonize the host [20]. In addition, complexes have been defined that consist of bacteria with varying degrees of pathogenicity [21]. Modern analyses have shown that this simplified representation is not sufficient. Complex interactions of the bacteria contained in the periodontal biofilm, as well as viruses and fungi, both with each other and with the host organism have been described. Individual components of the biofilm appear to play a more relevant role in the development of periodontitis than others [22,23]. In particular, the host’s inflammatory immune response to biofilm dysbiosis appears to be a major cause of the onset, establishment, and progression of periodontal destruction. Various mechanisms have been described in this regard [24]. In addition to the classic pro-inflammatory cytokines such as TNF-α, IL-1β, or IL-6 and the activation of TH1 and TH2 lymphocytes, the IL-23/IL-17 axis and thus the TH17 cells also appear to play an important role. They are also increasingly being discussed as a link to other chronic inflammatory diseases [25,26,27,28,29].

Periodontitis is associated with the occurrence of a wide range of general diseases, from cardiovascular diseases and diabetes mellitus to chronic inflammatory diseases such as rheumatoid arthritis, psoriasis, and chronic inflammatory bowel disease [15,30,31]. Various mechanisms that could explain the association are suspected [29]. Specific cytokine inhibitors, as used in the treatment of these diseases, could therefore also have an influence on the development of gingivitis and periodontitis. Little is known about this connection and in particular about the influence of targeted anti-cytokine therapy on the subgingival microbiota.

## 2. Materials and Methods

### 2.1. Study Cohort

**Figure 1:**
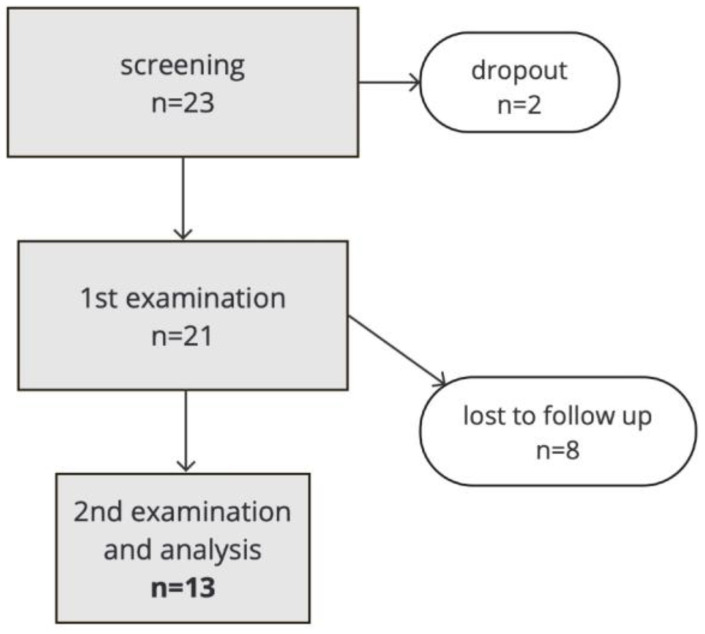
Flowchart of study cohort.

This monocentric, prospective, observational pilot study involved 21 patients with chronic inflammatory diseases, of which 8 patients dropped out after baseline (Figure 1). Patients received their first dose of biologic therapy according to guidelines and the decision of the interdisciplinary “inflammation conference“ during the study. They underwent dental examinations at two time points: the start of anti-cytokine therapy and approximately 14 weeks after initiation. 

The average follow-up time was 98.8 days (14 weeks). Recruitment was carried out in the years 2016–2017 from the patient population of the Biological Follow Up (BFU) Study of the Comprehensive Center for Inflammation Medicine (CCIM) of the University-Hospital Schleswig-Holstein (UKSH), Campus Kiel. Study participation was voluntary and occurred after written informed consent was obtained. The inclusion criteria were as follows:

- Diagnosed CID
- Upcoming targeted anti-cytokine therapy for the first time
- Participation in the Biological Follow Up Study of the CCIM
- Written informed consent

The exclusion criteria were defined as follows:

- Edentulousness
- Ongoing periodontal therapy
- Need for endocarditis prophylaxis

Participants were not selected based on whether they had been diagnosed with periodontitis. No information on their periodontal status was available prior to the start of the study. However, to prevent bias in the results, a questionnaire was used to ensure that the participants did not undergo periodontal therapy at any time during the study. In addition, detailed questions were asked about whether they had changed their oral hygiene habits in the period between the two examinations. 

Eight participants were lost to follow-up. Their data were not included in the longitudinal analyses, but the European Federation of Periodontology/American Academy of Periodontology (EFP/AAP) case definition was also applied for these participants except for one participant who withdrew consent. The remaining seven participants who were lost to follow-up did not provide any information about why they did not participate further. A priori power analysis gave a sample size calculation of 47 patients, with a power of 0.95. As the diseases and therapies described were rare even in our center at the time of the study, the pilot study was based on the data of 21 patients, with a power of 0.72 and a critical t of 1.72, calculated post hoc.

### 2.2. Serum Parameters and Dental Examination

The levels of C-reactive protein (CRP), leukocyte count, and platelet count were determined from fasting venous blood. Dental examinations were performed at the same time point in relation to the first intake of anti-cytokine therapy. The initial examination took place before the first dose (n = 6) or within 7 days after the first dose at the latest (n = 7). The follow-up examination took place after approximately 14 weeks and 16 weeks after the first administration of the anti-cytokine therapy at the latest. Examinations were always performed in a standardized manner according to the same scheme and by the same investigator.

Diagnoses of reduced periodontium were determined according to the consensus report on periodontal health and gingival diseases and conditions of an intact and reduced periodontium [32]. The given flowcharts of the EFP/AAP were used and suggested diagnoses of “healthy”, “healthy with reduced periodontium”, “gingivitis (localized/generalized)”, and “gingivitis (localized/generalized) with reduced periodontium or periodontitis”. For cases classified as periodontitis, we followed the 2018 AAP/EFP guidelines to classify each case as stage I, II, III, or IV, and the extent was classified as localized or generalized [33]. In detail, in the absence of radiographic bone loss, at least two non-adjacent teeth with interdental clinical attachment level (CAL) > 2 mm were used to assess a potential periodontitis case. All third molars or dental implants were excluded from diagnosis considerations. 

Sites with 4 mm pocket depth (PD) and no bleeding on probing (BOP) were considered healthy when the patient did not have PD > 4 mm. Sites with 4 mm PD and BOP were included as contributing to the diagnosis of periodontitis. CAL ≥ 5 mm represented stage III/IV, CAL of 3–4 mm represented stage II, and CAL of 2 mm represented stage I. CAL < 2 mm was considered healthy. The presence of PD ≥ 6 mm in at least 2 or more adjacent teeth and furcation involvement of class II or III was used to assess potential cases of stage III or IV. 

For the assessment of the extent of periodontitis, teeth with PD < 4 mm were included based on the assessed CAL. These teeth represented previously treated sites if there was history of periodontal treatment according to a questionnaire. If patients stated that there had been no previous periodontal treatment, these teeth were considered healthy. 

Probing depths were measured using a millimeter-scale periodontal probe (PCPUNC15, Hu-Friedy, Chicago, IL, USA) and six-point measurements (mesio-vestibular, vestibular, disto-vestibular, mesio-oral, oral, and disto-oral). Gingival recessions were determined using the same probe. For this purpose, the distance of the gingival margin to the enamel–cement interface was measured. From this, the CAL could be calculated by adding the probing depth and recession. If the enamel–cement interface was not accessible (e.g., due to a restoration), no value was recorded. 

To calculate the interdental CAL, the mid-surface measurements of the teeth (oral and vestibular) were not considered. Instead, the calculation was performed using only the mesio-vestibular, disto-vestibular, mesio-oral, and disto-oral readings. During the examination, bleeding caused by probing was noted, and the BOP index was calculated by dividing the number of bleeding points by the total number of sites probed. The degree of tooth mobility was determined as 0–3 according to Lindhe for each tooth [34].

Microbiome samples of the gingival crevicular fluid were obtained. The highest value of each participant’s sulcus depth was used to define the deepest periodontal pockets. Additionally, a clinically healthy pocket was defined as a probing depth under 2 mm and negative BOP. The microbiome samples were taken from one sulcus each at both examination times. 

To collect gingival crevicular fluid, sterile-packed paper tips (VDW, mtwo, absorbent paper tips, 29 mm) were inserted into the sulcus without pressure. They were left there for 5 s and then placed in a reaction tube. In case of visible contamination with blood, saliva, or pus, another sample was taken and labeled accordingly. This had to be performed a total of 4 times as 4 samples were likely contaminated with blood. All 4 samples were collected from the deepest measured pockets and taken at the second examination. Accordingly, additional sampling was performed in each case of pockets with a similar depth. The samples were immediately frozen at −80 °C.

### 2.3. Microbial Analysis

The samples were sequenced using 16S rRNA gene sequencing at the Institute of Molecular Biology (IKMB) at Kiel University. Automated DNA extraction was carried out first. The resulting solution containing the DNA was stored at −20 °C until further use. 

The v1–v2 region of the bacterial 16S rRNA gene was then amplified using polymerase chain reaction (PCR). For each sulcus sample, one primer pair consisting of forward and reverse and a different combination of two barcodes, each consisting of eight bases, was used. 

The primers were purchased from Metabion International AG (Planegg/Steinkirchen, Germany). This was followed by purification and normalization of the DNA concentration from the PCR products. Here, the SequalPrep Normalization Plate Kit (Thermo Fisher Scientific, Waltham, MA, USA) was used according to the manufacturer’s instructions. For efficient utilization of the MiSeq from Illumina and the corresponding MiSeq Reagent Kit v3 (2 × 300 bp, Ilumina, San Diego, CA, USA), samples were sequenced in one batch using pooling. Concentrations were measured using the Qubit Fluorometer (Thermo Fisher Scientific) and the Qubit dsDNA BR Assay Kit (Thermo Fisher Scientific) according to the manufacturer’s instructions. The sequences generated in this way were assigned to the respective samples if the barcode matched. Sequence segments complementary in both reading directions with an overlap of 250–300 bp were fused to generate longer reading segments.

Quality control was then performed using Q scores, a quality index of the MiSeq sequence data (FastQ format, Illumina). Sequences with a Q score below 30 in more than 5% of nucleotides were discarded according to a 0.1% probability of incorrect base calling. Chimeric sequences, which could result from the fusion of several fragments, were also removed.

Finally, OTUs (operational taxonomic units) were formed from the sequence data. Sequences with a homology of more than 97% were combined into an OTU.

The data were normalized to 10,000 sequences per sample to counteract artificial inflation. The taxonomic data were classified using the “SINTAX” algorithm based on the “RDP training Set 16” of the Ribosomal Database Project (RDP) database [35]. The abundances of the respective bacteria were determined at five different taxonomic levels: phylum (phylum), class (class), order (order), family (family) and genus (genus). 

The calculation and presentation of abundances was conducted at the phylum level. All further calculations were made at the highest available taxonomic level.

### 2.4. Statistical Analyses

Power and sample size were calculated with G*Power 3.1 [36,37]. R was used for statistical analyses and graphics [38]. Intraindividual α-diversities were calculated using the R packages VEGAN (https://cran.r-project.org/web/packages/vegan/index.html, access date 6 November 2020), ADE4 (https://cran.r-project.org/web/packages/ade4/index.html, access date 6 November 2020), and PHYLOSEQ (http://joey711.github.io/phyloseq/, access date 9 November 2020). The calculation was performed according to the distribution, which was tested using the Shapiro–Wilk test. A paired Student’s *t*-test was used when a normal distribution was present, and the Wilcoxon signed rank test was used when a normal distribution was not present. 

Interindividual β-diversity was determined as follows. Data from the available operational taxonomic units (OTUs) were merged with clinical information, information from the questionnaires, and taxonomic data to form an R-object called PHYLOSEQ [39]. Further analyses were also performed using the MICROBIOME (http://microbiome.github.io/microbiome, access date 9 November 2020, VEGAN (https://cran.r-project.org/web/packages/vegan/index.html, access date 6 November 2020), and GGPLOT2 (https://cran.rstudio.com/web/packages/ggplot2/index.html, access date 10 November 2020) packages. A permutational multivariate analysis of variance (PermANOVA) was performed using the adonis2 function of the VEGAN package [40]. The number of permutations was set to 10,000. Values of *p* < 0.05 were considered statistically significant. 

### 2.5. Ethics

The study was approved by the local ethics committee (AZ: A 156/03). Each proband gave informed consent before inclusion in the study. 

## 3. Results

### 3.1. Study Characteristics

In total, 21 participants were included. Eight participants were lost to follow-up, including one participant who withdrew consent. A total of 13 study participants completed the follow-up and were analyzed. This included nine women (69.2%) and four men (30.8%). The median age was 48 years, and the median body mass index was 26.6 kg/m^2^. The median sulcus depths in the deepest measured pockets of the test participants were 4.5 (4.0–5.0) mm in the first examination and 4.0 (4.0–5.0) mm in the second examination. The median sulcus depth of the clinically healthy pocket from which the microbiome probes were obtained was 2.0 (1.0–2.0) mm both before the start of anti-cytokine therapy and 15 weeks after. Four subjects had more than one, namely, exactly two known chronic inflammatory diseases. All four of these subjects have rheumatoid arthritis, three of them also have psoriasis or psoriatic arthritis, and one subject has ulcerative colitis.

At baseline, the median leukocyte value was 7.3 (6.5–8.3) × 10^3^/µL, which is within the normal range (4–10 × 10^3^/µL) (Table 1). However, it decreased slightly between the first and second examinations from 7.3 (6.5–8.3) to 6.5 (6.0–7.1). The mean CRP value decreased significantly (*p* = 0.01) from 2.3 (1.5–8.9) mg/L to 2.2 (1.1–2.9) mg/L (Table 1). 

Remission of the underlying CID was defined as a subjectively perceived improvement in the patient’s disease state, which was confirmed by a medical examination. In 9 of 13 cases, remission was detectable at the second examination. 

In the TNF-α group, symptoms improved in three of seven cases (43.9%), two additional cases experienced no significant change in disease activity, and one participant reported worsening. No information was available on the disease course of one participant in the TNF-α group. The remission rate for IL-17, IL-12/23, and IL-6 inhibitors was 100%, although it should be noted that the number of participants in these categories was lower than in the TNF-α group.

### 3.2. Microbiological Status of the Periodontal Sulcus

**Figure 2:**
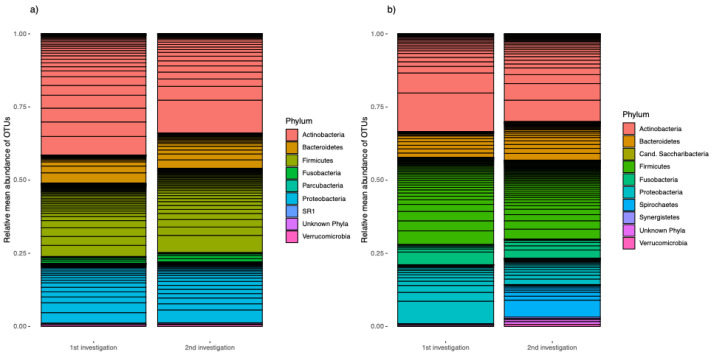
Relative abundances of the core measurable microbiome. (**a**) From clinically healthy and (**b**) from deepest pockets at the first and second examinations. The transverse lines show the OTUs that occur separately from each other.

Gingival crevicular fluid was taken from each patient at two sites: their deepest periodontal pocket and a healthy pocket that did not show attachment loss or BOP. At the second examination, samples were collected at the same sites. Analysis of these microbiome samples indicated that the clinically healthy periodontal pockets of all probands showed a relative mean abundance of Actinobacteria of 41.5% at the first examination (Figure 2a). Firmicutes accounted for the second most abundant proportion at 25.0%. Proteobacteria accounted for 20.5%, Bacteroidetes accounted for 9.7%, and Fusobacteria accounted for 2.3%. 

At 13 weeks after the start of specific anti-cytokine therapy, there was a decreased proportion of Actinobacteria (33.9%). Firmicutes represented 31.6%, Proteobacteria represented 20.8%, and Bacteroidetes represented 12.1%. Fusobacteria accounted for a relative mean abundance of 3.2%. The other phyla are shown in a graph, including unknown phyla, which made up less than 1% of the microbiome at both examinations.

The relative mean abundances of the samples from the deepest periodontal pockets are shown in Figure 2b. Actinobacteria made up the largest proportion of phyla (33.4% at the first examination and 30.0% at the second examination). Firmicutes made up the second most abundant proportion of phyla at both examination time points (29.7% and 26.8%, respectively). The proportion of Bacteroidetes increased from 8.8% at the first examination to 13.1% at the second one. Fusobacteria occurred with similar abundance (6.9% in the first examination and 6.6% in the second examination). Proteobacteria was significantly more abundant in the first examination (20.3%) than in the second examination (8.9%). 

Spirochetes were not found in any of the samples of the deepest measured pockets in the first examination, but in the second sampling, they accounted for 11.1%. The remaining observed phyla accounted for less than 1% with the exception of the unknown phyla at the second examination, which accounted for 1.9% (Figure 2b). 

### 3.3. α-Diversity under Anti-Cytokine Treatment

**Figure 3:**
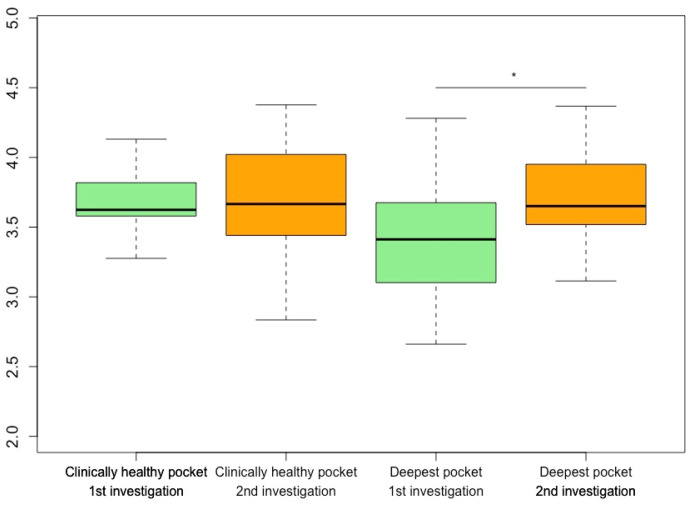
Alpha diversity (Shannon Index) for clinically healthy periodontal pockets (green) and deepest periodontal pockets (orange) of each patient at their first and second examinations * *p* < 0.05.

The analysis of α-diversity showed a significant increase in the Shannon Index of the deepest measured periodontal pockets between the first and second examinations (*p* = 0.039). With one exception, an increase in α-diversity in terms of the Shannon Index could be seen in all samples taken from the deepest periodontal pockets of all participants (Figure 3). An analysis of the α-diversity between drug groups showed no significant changes.

### 3.4. β-Diversity of Periodontal Pockets under Anti-Cytokine Therapy

**Figure 4:**
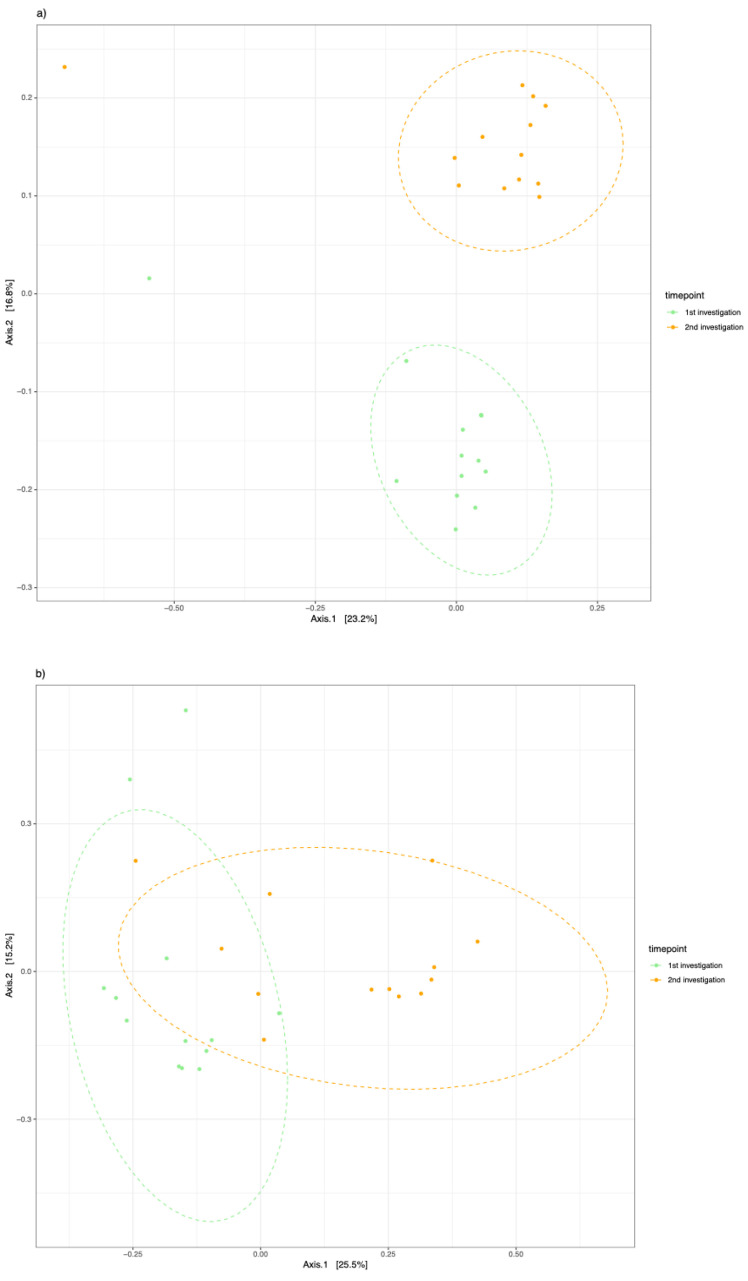
Principal coordinates analysis of the Jaccard Index for samples. (**a**) From clinically healthy pockets and (**b**) from clinically deepest measured pockets of the participants. The first study time point is shown in green, and the second time point after initiation of anti-cytokine therapy is shown in orange.

When distinguishing between clinically healthy pockets, principal coordinate analysis of the Jaccard index explained the variance in β-diversities of the clinically healthy pockets (*p* < 0.001; R^2^ = 11.7) and the deepest measured pockets of the participants (*p* < 0.001; R^2^ = 12.7). There was a clear difference in β-diversity after the initiation of anti-cytokine therapy, as displayed in Figure 4.

## 4. Discussion

Peddis et al. [41] conducted a systematic review of 35 publications addressing the interactions of anti-cytokine therapies and periodontitis. They included 15 non-randomized clinical trials, 12 in vivo animal models, and 7 case reports. The most studied disease was rheumatoid arthritis, followed by ankylosing spondylitis, psoriatic arthritis, systemic sclerosis, and Crohn’s disease. Most studies dealt with TNF-α blockers. The authors concluded that the evidence clearly supports the positive effects of anti-TNF-α therapy on periodontal health. Statements on other anti-cytokine approaches could not clearly be made. An examination of the oral microfilm is still lacking.

In the present study, we periodontally characterized participants with CID during the first 15 weeks of a targeted anti-cytokine therapy. The initial prevalence of periodontitis was above average. The median number of teeth with attachment loss decreased significantly. In patients with more than five teeth with attachment loss at baseline, the parameters of periodontal damage improved significantly. This improvement occurred without concomitant manual periodontal therapy or change in oral hygiene habits. 

Furthermore, changes in relative abundances of the subgingival microbiome at the phylum level were detected during specific anti-cytokine therapy. It is commonly known that the microbiome of a patient with periodontitis differs from that of a periodontally healthy patient [14]. In addition, differences in microbial composition between healthy and diseased sulci can be observed in intraindividual comparisons [32].

In the healthy samples and those from the deepest periodontal pockets, Actinobacteria were found to be the most abundant phylum, closely followed by Firmicutes. They accounted for about 1/3–1/4 of the phyla in each of the samples, and proportionally more Actinobacteria occurred in the clinically healthy sulci. Shi et al. [42] also described similar observations when examining samples in participants who were healthy, those with chronic periodontitis, and those with aggressive periodontitis (according to the 1999 classification). They described a dominance of Firmicutes, Actinobacteria, and Proteobacteria in the samples of periodontally healthy participants. Periodontitis showed fewer Actinobacteria and relative increases in Fusobacteria, Spirochetes, and Saccharibacteria [42]. 

It is interesting to note in this context that the samples that we examined were from participants without periodontitis, but some had increased pocket depths and CIDs. This suggests that there are early changes in the subgingival microbiome in such patients that might favor the development of periodontal inflammation. This would provide an explanation for the frequently described associations between periodontitis and chronic inflammatory diseases, such as rheumatoid arthritis [9,12], ankylosing spondylitis [43], psoriasis [44], and IBDs [11]. For IBDs, positive correlations between periodontal and intestinal inflammation have been described [45,46].

In one study, Atarashi et al. collected oral microbiome samples from patients with IBDs and transferred them into germ-free mice. They showed that the proliferation of TH1 cells was associated with the abundance of oral Klebsiella bacteria in the colon [47], suggesting a direct involvement of oral flora in intestinal inflammation. However, other explanations for associations of CIDs with periodontitis, such as shared genetic risk factors between rheumatoid arthritis and periodontitis [48] or altered parainflammatory conditions [49], have also been described.

However, it still needs to be discussed whether this improvement can be directly attributed to the given medication, possibly also through a change in the subgingival microfilm, or the improvement of the underlying CID. So far, the treatment of classical inflammatory targets, including IL-1 and TNF-α, have failed to meet expectations in periodontal therapy [50]. Our data imply that fundamental changes in the subgingival microbiota occur under targeted anti-cytokine therapy, leading to an improved periodontal status. 

Nevertheless, there are some limitations to this study, which was designed as a pilot study to develop hypotheses for further studies. The main limiting factors here are the small number of participants and the heterogeneous study group, with different systemic conditions, that are linked through inflammation. We characterized them in detail, but we did not have a healthy control group, and a study with a case-control design will be needed in the future. A future study direction would first have to allow a dedicated consideration of the individual entities and therapies separately from each other in order to be able to make reliable statements. However, we attempted to address this problem by using intra-individual microbiome samples from clinically healthy and less healthy tooth pockets. Furthermore, we tried to control for external influences such as changes in grooming habits or diet by distributing questionnaires. Nevertheless, biases due to the longitudinal study design are also conceivable.

## 5. Conclusions

Within the study limitations, the investigated data demonstrate a relationship between anti-cytokine treatment and the oral microbiome in patients with CIDs. In addition, anti-cytokine therapies could improve the microbiome diversity of the tooth pocket. This could be especially of interest for patients who have a higher tendency to develop periodontitis. However, it is still unclear whether periodontitis drives systemic inflammation, (or vice versa) or whether there is a mutual influence (which is more likely). Additional investigations are needed to analyze microbiome composition and metabolism directly in the tooth pocket and whether the systemic or local administration of specific biologicals can improve periodontal conditions in patients with CID and without CID.

## Figures and Tables

**Table 1:** Cohort characteristics, periodontal status, and serum parameters.

Variable	1st Examination	2nd Examination	*p*
Study cohort			
Probands, *n*	13	13	-
Women, *n* (%)	9 (69.2)	9 (69.2)	-
Age (years) [Median (IQR)]	48 (38–57)	48 (38–57)	1 ^a^
Active smokers, *n* (%)	5 (38.5)	5 (38.5)	1 ^b^
CID; patients, *n*			
Rheumatoid arthritis	7	7	-
Psoriasis/Psoriatic arthritis	5	5	-
IBD	3	3	-
Ankylosing spondylitis	2	2	-
Administered biologicals; patients, *n*			
Anti-TNF-α	6	7	-
Anti-IL-17	3	3	-
Anti-IL-12/IL-23	2	2	-
Anti-IL-6	1	1	-
Periodontal status [Median (IQR)]			
Bleeding on probing	16.1 (9.3–21.0)	13.0 (7.3–30.4)	0.6 ^a^
PD	2.0 (1.9–2.3)	2.0 (1.9–2.2)	0.5 ^a^
DMFT	17 (14–20)	17 (14–20)	1 ^a^
Microbiome sample collection [Median (IQR)]			
Healthy pocket, PD	2.0 (1.0–2.0)	2.0 (1.0–2.0)	0.3 ^c^
Deepest pocket, PD	4.5 (4.0–5.0)	4.0 (4.0–5.0)	0.2 ^c^
Serum parameters [Median (IQR)]			
CRP [mg/L]	2.3 (1.5–8.9)	2.2 (1.1–2.9)	0.01 ^c^ *
WBC count [×10^3^/μL]	7.3 (6.5–8.3)	6.5 (6.0–7.1)	0.21 ^a^

Abbreviations: IQR, interquartile range; CID, chronic inflammatory disease; IBD, inflammatory bowel disease (Crohn’s disease and ulcerative colitis); PD, pocket depth; DMFT, decayed missing filled teeth; CRP, C-reactive protein; WBC, white blood cell. Note: ^a^ Paired Student’s *t*-test; ^b^ $\chi^{2}$-Test; and ^c^ Wilcoxon-signed-rank test; * Statistically significant (*p* < 0.05).

## Data Availability

All samples and information on their corresponding phenotypes were obtained from the PopGen Biobank (Schleswig-Holstein, Germany) and can be accessed through a Material Data Access Form. Information about the Material Data Access Form and how to apply can be found at “http://www.uksh.de/p2n/Information+for+Researchers.html” accessed on 23 August 2024.
